# Editorial Note: Parental non-involvement strategy for handling sibling conflict on social avoidance in migrant children: Chain mediation of sibling conflict and parent-child conflict

**DOI:** 10.1371/journal.pone.0331103

**Published:** 2025-08-26

**Authors:** The PLoS One Editors

This article [1] was identified as one of a series of submissions for which we have concerns that the peer review did not meet the expected standards for *PLOS One*. We regret that the issues were not identified and addressed prior to the article’s publication.

This note is not intended to question the quality of the article or contributions of the authors to the review process.

## References

[1] Yue Y, Lu K, Ye D. Parental non-involvement strategy for handling sibling conflict on social avoidance in migrant children: Chain mediation of sibling conflict and parent-child conflict. PLoS One. 2024;19(9):e0308561. doi: 10.1371/journal.pone.0308561 39255279 PMC11386452

